# APCAP - activated protein C in acute pancreatitis: a double-blind randomized human pilot trial

**DOI:** 10.1186/cc9203

**Published:** 2010-07-27

**Authors:** Ville Pettilä, Lea Kyhälä, Marja-Leena Kylänpää, Ari Leppäniemi, Minna Tallgren, Antti Markkola, Pauli Puolakkainen, Heikki Repo, Esko Kemppainen

**Affiliations:** 1 Department of Anesthesia and Intensive Care Medicine, Helsinki University Central Hospital, Haartmaninkatu 2, Helsinki, 00029, Finland; 2 Department of Epidemiology and Preventive Medicine, Australian and New Zealand Intensive Care Research Centre, Monash University, 99 Commercial Road, Melbourne, VIC 3004, Australia; 3 Department of Surgery, Helsinki University Central Hospital, Haartmaninkatu 2, Helsinki, 00029, Finland; 4 Department of Radiology, Helsinki University Central Hospital, Haartmaninkatu 2, Helsinki, 00029, Finland; 5 Department of Medicine, University of Helsinki, Haartmaninkatu 2, Helsinki, 00029, Finland; 6 Department of Bacteriology and Immunology, University of Helsinki, Haartmaninkatu 2, Helsinki, 00029, Finland

## Abstract

**Introduction:**

Previous human studies have shown low activity of protein C (APC) in severe acute pancreatitis (SAP). This, together with the findings in animal models, suggests that activated protein C (APC) may protect against pancreatic injury and ameliorate the disease. We, therefore, evaluated its effect on multiple organ dysfunction (MOD) measured by the SOFA (Sequential Organ Failure Assessment) and on organ-failure-free days, and the safety of APC in SAP.

**Methods:**

A prospective double blind randomized pilot study was use. The study occurred in one university hospital tertiary intensive care unit (ICU) with eight beds. The patients were chosen according to the following inclusion criteria: 1) Those admitted to the hospital < 96 h from the onset of pain, 2) Those who had a three-fold increase in serum amylase over normal upper range or/and in whom computed tomography (CT) verification of SAP was noted, 3) Those who had one or more organ dysfunction (OD), and 4) Those in whom less than 48 hours had passed since their first OD. Of a total of 215 adult patients with SAP screened between June 2003 and August 2007, 158 fulfilled the study inclusion criteria. After exclusions 32 patients were randomized to the study. The intervention consisted of APC (*N *= 16) administered intravenously for 96 hours with a dose of 24 μg/kg/hour or placebo (*N *= 16) with a similar infusion rate. The sample size for the study was calculated according to the primary end-point: the change in SOFA during study drug infusion (Days 0 and 5). Comparisons between the study groups were performed using patient-related changes and calculation of difference in means (DIM, 95% CIs) and regarding categorical variables with Fisher's exact test. For all comparisons *P *< 0.05 was considered significant.

**Results:**

No serious bleeding was detected clinically or by CT scans in either group. No significant difference in SOFA score change between the APC and placebo groups was found (difference in means (DIM) +2.3, 95% CI -0.7 to +5.3). Treatment with APC was associated with an increase in serum levels of both total and conjugated bilirubin. No differences in ventilator-free days, in renal replacement therapy-free days, in vasopressor-free days, or in days alive outside the hospital were detected.

**Conclusions:**

No serious bleeding or differences in the evolution of MOD were detected between APC and the placebo. Instead we found an increase in serum bilirubin in the APC group compared to the placebo group in patients with SAP.

**Trial registration:**

ClinicalTrials.gov NCT01017107.

## Introduction

Regardless of achievements in critical care the overwhelming inflammatory response [1] in patients with severe acute pancreatitis (SAP) still leads to multiple organ dysfunction (MOD) in over 60% [2] and to hospital death in 6 to 47% [2,3] of cases. In MOD patients with at least two failing organs, according to the Sequential Organ Failure Assessment (SOFA) [4], the hospital mortality rates may be as high as 50 to 91% [2]. Furthermore, systemic activation of the coagulation system commonly occurs in over half of the critically ill patients with SAP [2]. In rabbits an immediate activation of protein C (PC) is a specific characteristic of hemostatic activation in SAP [5]. We have also previously shown that plasma samples in SAP drawn before MOD showed low PC levels and high endogenous activated protein C (APC) to PC ratios [6].

Exogenous recombinant human activated protein C (drotrecogin alpha activated) has been shown to significantly reduce myeloperoxidase levels in acute experimental pancreatitis in rats [7]. Based on an experimental model inhibition of expression of pancreatic p38 MAPK and JNK and up-regulation of ERK1/2 expression by APC treatment may theoretically protect against pancreatic injury and ameliorate the disease [8]. In addition, APC has improved the severity of pancreatic tissue histology, and decreased the super-infection rate and serum markers of inflammation during the course of experimental acute necrotizing pancreatitis [9].

Unlike other pharmacologic treatments APC has been shown to reduce 28-day mortality by 6.1% in a large randomized controlled trial (RCT) in severe sepsis [10]. Thereafter, the benefits of the treatment have been questioned with regard to the excess bleeding [11-13], and confirmatory RCTs are ongoing to confirm or refute the benefit in septic shock [14]. Based on the positive RCT in severe sepsis [10] and the above mentioned laboratory studies in SAP it has been suggested that APC may also be beneficial in SAP [15]. However, no human RCT focusing on APC in SAP is available to date. Thus, no evidence exists to justify the use of APC in the early stages of SAP with specific concerns of risk of hemorrhage in patients with prolonged pancreatitis and pancreatic necrosis [16].

Accordingly, we conducted a prospective randomized double-blind pilot study in 32 patients with SAP (and without severe sepsis). We hypothesized that APC would be associated with decrease in SOFA during the treatment and also aimed to assess organ-failure free days to detect potential signs of benefit of APC in SAP for further hypothesis generating purposes. We also recorded the number of serious cases of bleeding in both study groups.

## Materials and methods

The ethics committees of the Helsinki University Central Hospital approved the study. All patients or their next of kin gave an informed consent for participation in the study. The study protocol was registered in ClinicalTrials.gov (NCT01017107).

### Study population

**Figure 1:**
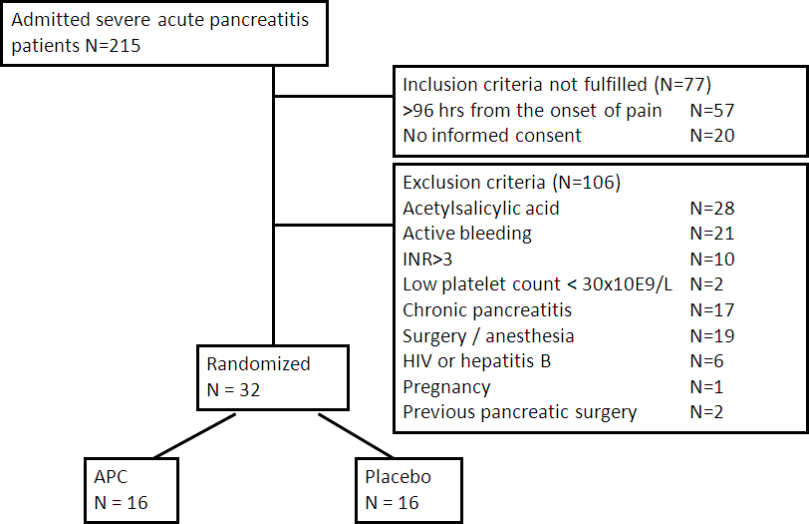
**Flow-chart of the study**.

A total of 215 admissions with SAP [17] were screened for the study in one tertiary care university hospital between June 2003 and August 2007. Of the 215, 158 fulfilled the study inclusion criteria: 1) admitted to the hospital < 96 h from the onset of pain, 2) a three-fold increase in serum amylase (IU/L) over normal upper range or/and verification of SAP in computer tomography (CT) [17], 3) at least one organ dysfunction (OD) defined as organ specific SOFA score of at least three of four, and 4) < 48 hours from the first OD [18]. Finally 32 patients with SAP were randomized to receive either APC (drotrecogin alpha activated) or 0.9% physiologic saline [10,18] as a placebo (*N *= 16). For study flow-chart and reasons for exclusion see Figure 1.

### Intervention

After informed consent the baseline SOFA score was registered. The randomization was performed by an independent statistician using block randomization with variable block size. The investigators were blinded for the study treatment and the block size. To increase the comparability of study groups regarding MOD at baseline, stratification was performed according to SOFA score: patients with SOFA scores from 3 to 8, or at least 9 were randomized separately to the APC or placebo group. The code for study medication was concealed using sealed envelopes. An independent study nurse from another ICU, not involved in the treatment of the study patients, opened the sealed envelope at the request of the investigator, and prepared either the APC and placebo infusions for the ICU. APC was administrated intravenously for 96 hours with a fixed dose of 24 μg/kg/hour [10,18] or placebo with the same rate and similar infusion bag with black infusion lines. The investigators and the treating personnel remained double-blinded to the randomized study drug until the study database was finalized. The standard treatment was administered according to a written protocol including early enteral nutrition; use of pulmonary artery catheter, colloid and crystalloid fluid administration, norepinephrine and/or dobutamine infusions in cardiovascular failure; lung-protective ventilation; sedation with propofol and/or midatzolam, and/or fentanyl infusion for pain. The only indication for early surgical treatment during the first three weeks was increased intra-abdominal pressure (> 25 H2O cm) with a new organ dysfunction. The standard treatment remained unchanged during the study and was in close agreement with the international guidelines [19] except that we used cefuroxime (first week) and, meropenem thereafter for infection prophylaxis (which is no longer recommended).

### Data collection

Demographic data, co-morbidities, APACHE II (Acute Physiology and Chronic Health Evaluation, SOFA score [4], treatment data, ICU and hospital length of stay (LOS), and hospital mortality were registered.

### Laboratory measurements

The primary safety endpoint was the number of incidents of bleeding, and the primary efficacy endpoint was the change in SOFA between the start of the study drug (Day 0) and Day 5 [20,21]. Laboratory measurements for SOFA (serum creatinine, serum bilirubin, platelet count and blood gas analysis for PaO2/FiO2-ratio) were performed. After primary analysis of serum total bilirubin (μmol/L), a *post hoc *secondary analysis was performed to detect possible patient-related changes in plasma hemoglobin (p-Hb, mg/L), serum conjugated bilirubin (μmol/L), serum pre-albumin (mg/L), serum disialotransferrine (DST, %) and serum gamma-glutamyl-transferase (y-GT, U/L) between the study groups. All laboratory tests were performed using routine laboratory methods (Helsinki University Central Hospital Laboratory).

Secondary endpoints were: Organ-failure-free days alive (ventilator-free days [22], renal replacement therapy-free days, vasopressor-free days), days alive outside hospital in 60 days, and changes in other laboratory values during the first five days.

### Radiologic assessment

An abdominal CT-scan was performed on an MX QUAD (Phillips, Medical Systems, Eindhoven, the Netherlands) before the administration of the study drug and seven days thereafter to reveal the severity of disease before the study drug administration and to detect possible complications during the treatment. Severity of acute pancreatitis was scored from 0 to 10 by a specialist in radiology (AM) who was blinded for the treatment. A classification by Mortele and colleagues [23] was used for the evaluation.

### Statistical methods

The sample size for the study was calculated according to the primary end-point: the change in SOFA [4]. Based on our previous study [2] we assumed that critically ill SAP patients would have an average admission SOFA score of 7 and that the mean (SD) increase would be 1 (± 3) points in the placebo group. Calculations according to established methods revealed that a sample of 16 patients per group would allow us to detect a clinically meaningful three-point difference in change of SOFA score (7 to 8 in placebo group vs. 7 to 5 in APC group with an estimated SD of 3.0 and with *P *< 0.05 and a power of 80%). The sample size was also assumed to be adequate for testing the differences in patient-related change in laboratory parameters in this pilot trial (no preliminary data were available for calculations). The study was not powered to detect any differences in mortality.

Data are presented as means + SD or numbers of patients, as appropriate. According to the assumption that a considerable number of study patients would die early in the study, and the possibility that the chosen sample size would carry the risk of non-comparability at the baseline, we decided to perform the statistical comparison using patient-related changes (before and after the study drug, Days 0 and 5) in laboratory values, modified CT and SOFA scores [20], and to use a short time-period (related to duration of study drug infusion) for the analyses. Comparisons between the study groups were performed using these changes and the calculation of differences in means (DIM (95% CIs)) according to evidence-based medicine guidelines for randomized studies [24], and regarding categorical variables with Fisher's exact test. The normal distribution of study variables was confirmed using Kolmogorov-Smirnov test. For all comparisons *P *< 0.05 was considered significant. The analyses were performed using the SPSS 15.0 software (SPSS, Chicago, IL, USA).

## Results

### Demographics

The study consisted of 32 adult patients with SAP. The demographics of study groups are presented in Table 1. All randomized patients received the randomized treatment with no un-blinding of the code or cessation of the infusion due to safety reasons. In the APC group 6, 0, 3, 5 and 2 patients had 1, 2, 3, 4 and 5 ODs according to the SOFA score at baseline compared to 7, 1, 3, 4 and 1 patients in the placebo group, respectively.

**Table 1:** Demographic data of the study patients with severe acute pancreatitis (SAP) at baseline

	Activated protein CN = 16	Placebo *N *= 16	*P*-value
Male/female	15/1	14/2	1.0
Etiology			
Alcohol/biliary	16/0	15/1	0.5
Age (years)	47.0 (± 8.1)	43.9 (± 11.0)	0.62
Weight (kg)	90.1 (± 19.1)	88.3 (± 15.0)	0.72
Serum amylase (IU/L)	1460 (± 1079)	703 (± 445)	0.03*
SOFA score	6.5 (± 4.0)	6.3 (± 3.1)	0.74
APACHE II score	17.6 (± 8.5)	14.0 (± 5.0)	0.26

Data are presented as numbers or mean (± SD). APACHE II, Acute Physiology and Chronic Health Evaluation II score. SOFA, Sequential Organ Failure Assessment in at baseline.

### Safety data

One patient died after receiving 13 hrs of APC infusion, and another non-survivor had a laparotomy at 41 hrs of APC infusion. All the other patients in the APC group received APC infusion targeted to 96 hrs with temporarily cessations for invasive procedures (range of duration 91 to 97 hrs). No significant bleeding was detected in the autopsies. No serious bleeding complications were registered clinically or by CT scan in either group (incidence 0%, 95% CI 0 to 18%, DIM 95% CI -17 to +17%). Minor bleeding (from mouth, nose, and urinary bleeding) occurred in four patients in the APC and in two patients in the placebo group (*P *= NS). The total number of administered red blood cell units during the study drug infusion did not differ between the study groups (seven in APC vs. eight in placebo group, *P *= NS).

The study revealed no difference in the severity of SAP between the groups by modified CT score at the baseline (5.8 in the APC group vs. 5.3 in controls). Patient-related changes in the modified CT score and in laboratory values are presented in Table 2. Treatment with APC was associated with an increase in serum bilirubin (DIM 28.4 mmol/L (95% CI 3.6 to 53.1)), and serum conjugated bilirubin (DIM 25 mmol/L (5.6 to 44.4), respectively). For three patients in APC group and four patients in the placebo group the missing Day 5 bilirubin value was substituted by the value of the preceding day. No significant differences in other laboratory variables were found (Table 2). No differences between the study groups were detected in the change of serum DST or gamma-GT (data not shown).

**Table 2:** Safety and efficacy variables of the study presented as mean and difference in means (95% confidence interval)

	N	APC		Placebo	Difference in means between the study groups (95% CI)
Delta SOFA score	16	1.8 (4.3)	16	-0.6 (4.1)	2.3 (-0.7 to 5.2)
Delta-non-hepatic SOFA	16	1.9 (4.1)	16	-0.2 (3.9)	2.1 (-0.8 to 4.9)
Ventilator free days	16	10.4 (9.4)	16	10.4 (11.5)	0 (-7.4 to 7.4)
Renal replacement therapy-free days	16	52.7 (12.7)	16	55.3 (9.3)	-2.6 (-10.5 to 5.3)
Vasopressor-free days	16	52.8 (7.1)	16	55.1 (6.0)	-2.6 (-5.9 to 2.3)
Alive outside hospital - days	16	17.1 (32.5)	16	34.4 (13.0)	-17.4 (-0.1 to -34.9)
Delta modified CT score	11	0.5 (2.0)	11	0.5 (1.8)	0 (-1.6 to 1.6)
Delta serum bilirubin, total (μmol/L)	16	18 (46.4)	16	-10 (17.0)	28 (3.6 to 53.1) *
Delta serum bilirubin, conjugated (μmol/L)	13	14 (31.9)	12	-11 (11.1)	25 (5.6 to 44.4) *
Delta serum creatinine (μmol/L)	16	-37 (84)	16	13 (86)	-50 (-110 to 10)
Delta platelet count (× 10^9^/L)	16	32 (52)	16	35 (55)	-3 (-40 to 34)
Delta plasma hemoglobin mg/L	13	-226 (499.5)	16	-65 (83.2)	-161 (-413 to 92.0)
Delta serum pre-albumin mg/L	13	-50 (49.7)	12	-69 (55.2)	19 (-23.0 to 61.0)

Organ-dysfunction-free days calculated for 60 days.

*significant differences; CI, confidence interval.

### Efficacy data

During the treatment (from Day 0 to Day 5) the mean (± SD, range) SOFA score changed from 6.5 (± 4.0, 2 to 14) to 8.2 (± 5.0, 0 to 16) in the APC group and from 6.3 (± 3.1, 3 to 11) to 5.7 (± 4.6, 0 to 16) in the placebo group (DIM 2.3, 95% CI -0.7 to 5.2). The range for individual change in SOFA score was -7 to +10 points. The median of the maximal SOFA score during the ICU stay was 10 in both groups. After detecting a difference in change of bilirubin, a *post hoc *analysis of non-hepatic-SOFA score was performed (see Table 2). Of the 32 patients, 21 (66%) needed mechanical ventilation, and the median length of the mechanical ventilation was eight days (range 0 to 39 days). Twelve (38%) needed dialysis because of renal failure and 20 of 32 (63%) needed vasopressor treatment during their stay at the ICU. The median duration of ICU stay was 11 days (range 0 to 43 days). The median hospital stay was 23 days (range 2 to 140 days). No differences in ventilator free days, in renal replacement therapy-free days, in vasopressor-free days, or in days alive outside the hospital, were detected (Table 2).

The 30-day mortality (not an endpoint) in the APC group was 3 of 16 compared to none of 16 in the placebo group (ARR -19%, 95% CIs -38% to 0). The non-survivors had pre-randomization SOFA scores of 10, 13 and 14 indicating MOD, which was also the autopsy-confirmed cause of all deaths (on Days 4, 11 and 14). A total of three patients underwent laparotomy, two of them (one non-survivor) in the APC group.

## Discussion

We undertook a single-center double-blind pilot RCT in 32 patients with severe acute pancreatitis, and found that patients randomized to receive APC had no more bleedings compared to the placebo group. In addition, our study revealed no beneficial effect of APC on MOD. The serum total and conjugated bilirubin levels increased in the APC group compared to the placebo group.

In our study we found no severe bleeding associated with APC treatment. The main purpose of this pilot study was to focus on this issue, because the increased risk of bleeding with APC has raised concerns [11]. The data from randomized trials in severe sepsis patients have shown a slightly increased risk of death from serious bleeding with APC (0.7% vs. 0.2% with placebo) and a rate of 2.7% to 5.5% of serious bleeding in surgical patients during the APC infusion [13]. Given that the patients with SAP are supposed to be prone to thrombocytopenia [2], disseminated intravascular coagulation [25] and intra-abdominal bleedings these concerns seem to be justified. However, based on *post hoc *subgroup analysis of patients with severe sepsis and acute pancreatitis in the PROWESS trial [10], APC has been recommended for SAP patients with severe sepsis bearing in mind the concern of retroperitoneal haemorrage [19]. Fortunately, none of our 16 patients with APC had retroperitoneal haemorrhage or other serious bleeding in CT scans and in none of the three expired patients in this group autopsy could verify any bleeding to be cause of the fatal outcome. Of note, however, the absence of serious bleeding in both groups should be seen in the light of our study design excluding patients with decreased platelet count and the low number of patients with operative treatment (2 of 16 in the APC group).

Our study detected a significant increase in serum bilirubin levels in the APC group, both in total and conjugated values. The reason for this increase remained unsolved, because no difference in patient-related plasma hemoglobin levels, serum gamma-GT, pre-albumin, or in DST was detected. In the literature, early bilirubin increase during Days 1 to 7 has been detected previously also in severe sepsis patients treated with APC [26]. Despite the comparable baseline hepatic SOFA both the averaged hepatic SOFA scores for Days 1 to 7 and the prevalence of hepatic organ dysfunction were increased in APC group compared to placebo in the PROWESS trial [26]. Our results, which showed increase in total bilirubin levels with APC, are in agreement with the previous findings in a different patient group of severe sepsis.

This pilot study was underpowered to detect small differences in MOD assessed by SOFA score. Given the wide range in individual change in SOFA (-7 to +10 SOFA points), our study had a moderate chance to detect a three-point decrease in patient-related change in SOFA score during the first five days. However, in this pilot we endeavored to seek a trend for hypothesis generating purposes. Regrettably, no such positive trend in SOFA score was detected in either days free of organ dysfunction or in a change of SOFA score. On the contrary, we detected a non-significant difference in means of 2.3 in SOFA score in advantage of placebo, a trend not explained by the bilirubin changes. To sum up, although the results of pilot studies should be considered with caution, we could not detect any beneficial sign to justify a larger RCT with APC in SAP, at least with similar inclusion criteria, in alcohol-induced acute pancreatitis (> 90%), and with a similar timing, dose and duration of APC treatment.

No previous RCT in humans evaluating the safety and efficacy of APC in SAP has been conducted. Only a few cases of acute pancreatitis accompanied by severe sepsis have been treated with APC [10,27]. Previous animal models of SAP have suggested decreased inflammation, up-regulation of expressions of endothelial cell protein C receptor and thrombomodulin [28], and improved survival with APC in rats [7], although reports have been contradictory [29]. Other proposed mechanisms for possible benefits in SAP have been the regulation of mitogen-activated kinases [8], a decrease in leukocyte-endothelial interaction [30], improved intestinal microcirculation [31], and a decrease in infection of mesenteric lymph nodes [9]. In addition, APC has been shown to modulate the systematic inflammatory response by inhibition of tumor necrosis factor and nuclear factor-kappa-B (NF-κB) release [32], among other proposed beneficial mechanisms in severe inflammatory disorders [33]. Given that previous studies have detected lower levels of APC and AT III in non-survivors [34] and low endogenous PC levels and high APC:PC ratios are found in MOD patients in human SAP [6], we previously agreed to a biologically plausible rationale for the use of APC in SAP. In keeping with this, APC has been assessed as a potential treatment option for SAP [15,16]. Accordingly, we undertook a randomized pilot trial but found no signs of benefits of APC in SAP.

Our study has several strengths. The study was a double-blinded trial, the concomitant treatment did not change during the study period, the disease severity of SAP in terms of SOFA and APACHE II scores was comparable to our previous observational study [2], and patients not included in the study were registered according to the CONSORT guidelines [35]. In addition, the placebo group received the best available standard treatment. Our study thus avoided the potential bias caused by under-treatment of the placebo group [36] and the mortality in the placebo group was in the range of best available practice (< 20%). We also used the same dose of APC as in the two first large trials in severe sepsis [10,18].

Our study also has some limitations. First, the groups were not totally comparable after randomization. The patients in the APC group had a higher serum amylase level (although not reflecting the severity of SAP), and those three patients who died of MOD already had high SOFA scores (10 to 14) before the study drug infusion. Extrapolated from the severe sepsis trials we reasoned that the most severely ill SAP patients might also gain the most possible benefit and, therefore, did not exclude patients with severe MOD at baseline. It could be argued that some of our patients were too sick or too early or late in the course of their disease to have any advantage of APC. Compared to a recent study of 55 patients with acute pancreatitis (with a 30-day mortality of 27%) [37], the patients in this study had higher baseline SOFA (6.4 vs. 5.8) and APACHE II (15.8 vs. 12.2) scores. Furthermore, we used only patient-related changes in our pre-defined analysis to overcome the problem with slight trends to differences at baseline. In addition, SAP patients with high SOFA scores have the possibility to survive [2] as also shown in the placebo group (SOFA range up to 16) in the present study. However, although the median of maximum SOFA scores was 10 in both treatment groups indicating MOD, due to a limited sample size we cannot definitely rule out the benefit of APC in SAP patients with even higher baseline SOFA scores and more severe MOD. Second, the choice of a similar timing, dose and duration of APC treatment as in severe sepsis trials may be criticized. It is possible that patients with SAP would need a higher dose due to an overwhelming inflammatory response. For a pilot study without any previous human studies, we found it safer to use the dose previously used in RCTs of severe sepsis. Third, the outcome in the placebo group was better than expected which may have caused bias to assessment of the SOFA scores. Fourth, due to sample size we can only conclude that we could exclude a difference in incidence of serious bleeding exceeding 17%. A larger sample size is needed to exclude possible smaller differences in the safety of APC in SAP. Fifth, an alternative study approach could have been to include only a subgroup of SAP patients with low PC and/or antithrombin levels at baseline. Finally, we evaluated the change in SOFA in five days [20], which may be too short a time to detect the difference in MOD. However, no long-term outcome measure showed a benefit either.

## Conclusions

We conclude that no serious incidents of bleeding were detected in severe acute pancreatitis patients who received APC. In addition, we found no differences in the evolution of MOD or in organ-failure-free days between the study groups. In contrast, the study revealed increases in serum total and conjugated bilirubin levels in SAP treated with APC compared to the placebo.

## Key messages

• Previous evidence suggests that activated protein C (APC) may protect against pancreatic injury.

• In a randomized pilot human clinical trial comprising 32 patients with severe acute pancreatitis we found no difference in serious bleeding or development of multiple organ dysfunction between APC and the placebo.

• Serum bilirubin levels increased in the APC group.

## Abbreviations

AP: acute pancreatitis; APACHE: Acute Physiology and Chronic Health Evaluation; APC: activated protein C; CT: computed tomography; DIM: difference in means; DST: disialotransferrine; ICU: intensive care unit; LOS: length of stay; MOD: multiple organ dysfunction; RCT: randomized controlled trial; SAP: severe acute pancreatitis; SOFA: Sequential Organ Failure Assessment.

## Competing interests

This study was supported by EVO-grant TYH 5226 from Helsinki University Hospital. Eli Lilly in part provided the study drug for this investigator-initiated study, but had no influence on the study design, data analysis or report. The investigators take full responsibility of the integrity and content of this paper.

## Authors' contributions

VP, LK and MLK executed the study and drafted the manuscript. VP, MLK, AL, MT, PP, HR and EK participated in the original design and coordination of the study, and in writing the original protocol. VP, LK and MLK analyzed the data. VP, LK, MLK, AL, MT, PP, HR and EK assisted in drafting the manuscript. AM analyzed the CT scans. All authors read and approved the final manuscript.
